# Supplementary material for: Structural and Thermodynamic Insights into Dimerization Interfaces of Drosophila Glutathione Transferases
Source: Biomolecules. 2024 Jun 26;14(7):758. doi: 10.3390/biom14070758 (PMC11274453; doi:10.3390/biom14070758)
Supplement: Supplementary file 1 [file biomolecules-14-00758-s001.zip › biomolecules-3050655-supplementary.pdf]

Supplemental

Structural and Thermodynamic Insights into Dimerization Interfaces of Drosophila glutathione transferases

Mathieu Schwartz[1], Nicolas Petiot[2], Jeanne Chaloyard[1], Véronique Senty-Segault[1], Frédéric Lirussi[3,4,5], Patrick Senet[2], Adrien Nicolai[2], Jean-Marie Heydel[1], Francis Canon[6], Sanjiv Sonkaria[7], Varsha Khare[7], Claude Didierjean[8] and Fabrice Neiers[1,*]

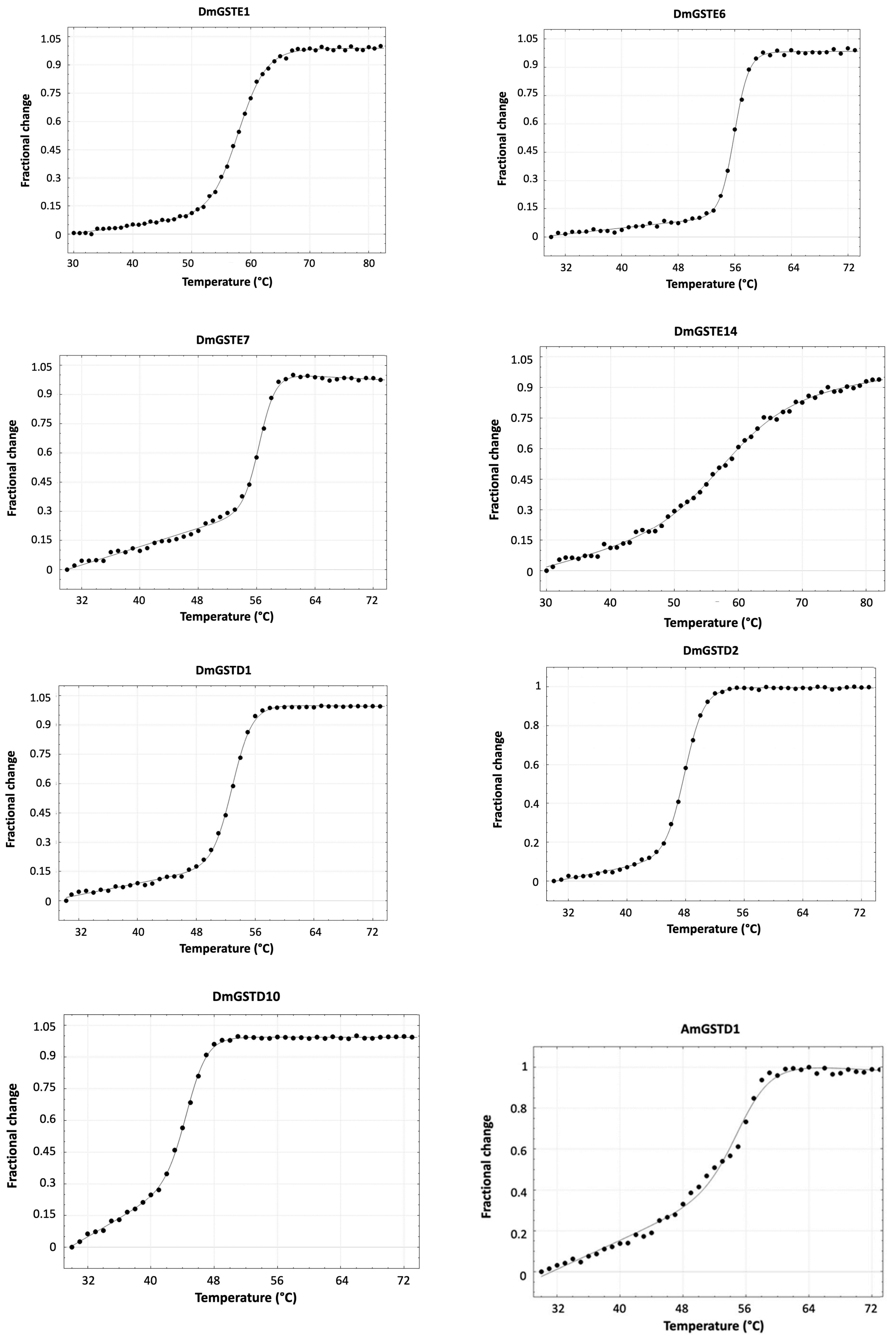

Supp Figure 1: Temperature denaturation of the tested insect GSTs followed by the normalized fractional change at 220 nm measured from CD experiments. The data were fitted with the CDPAL software.
